# Efficacy of Computerized Tomography-Guided Core Biopsy in Identifying the Subtypes of Lung Adenocarcinoma: An Observational Perspective From Pakistan

**DOI:** 10.7759/cureus.57337

**Published:** 2024-03-31

**Authors:** Babar Yasin, Hasan Saeed, Muhammad Awais Ahmad, Sara Najam, Mehwish Niazi, Humza Tariq, Allah Yar Yahya Khan, Shoaib Khaliq, Syeda Gul e Zehra Zaidi, Haseeb Mehmood Qadri

**Affiliations:** 1 Histopathology, Aznostics - The Diagnostic Center, Lahore, PAK; 2 Histopathology, Shifa International Hospital Islamabad, Islamabad, PAK; 3 Histopathology, Akhtar Saeed Medical and Dental College, Lahore, PAK; 4 Internal Medicine, Jinnah Hospital Lahore, Lahore, PAK; 5 Histopathology, Fatima Memorial Hospital College of Medicine and Dentistry, Lahore, PAK; 6 Surgery, Lahore General Hospital, Lahore, PAK; 7 Surgery, Farooq Hospital, Lahore, PAK; 8 Surgery, Jinnah Hospital Lahore, Lahore, PAK

**Keywords:** core biopsy, ct-guided biopsy, lung biopsy, pakistan, subtype, adenocarcinoma, lung cancer

## Abstract

Background

Lung carcinoma is a leading cause of death worldwide. Histological subtype of lung adenocarcinoma is an important indicator of patient's outcome as it is helpful in surgical planning and guidance of prognosis.

Objective

To determine the diagnostic efficacy of computerized tomography-guided core needle biopsy (CNB) in identifying the histopathological subtype of lung adenocarcinoma.

Methods and materials

This is a retrospective, descriptive study including clinical data of 73 patients irrespective of their age and gender, who underwent computerized tomography-guided CNB for lung masses at the Department of Pathology, Aznostics - the Diagnostic Centre, Lahore, Pakistan from January 01, 2019 to June 30, 2023. Data collected was analyzed via Google Form (Google Inc., Mountainview, CA) and Statistical Package for Social Sciences (IBM SPSS Statistics for Windows, Version 24, released 2016; IBM Corp., Armonk, New York, United States) and was sent to statistician for descriptive analysis. Categorical data was used for calculating frequency and percentage, while continuous data was computed as mean and standard deviation.

Results

Seventy-three patients with adenocarcinoma underwent pulmonary biopsy. The mean age of included patients was 64.88 ± 11.39 year with a male predominance of 61.64%. Upper lobe was commonly affected by adenocarcinoma lung in 57.53% patients and 58.90% cases involved the right lung. The most common subtype was acinar with 51.65% followed by solid with 17.58% cases. Computerized tomography-guided CNB showed a diagnostic yield of 75.34% and identified histological subtypes of lung adenocarcinoma in 55 cases.

Conclusion

Computerized tomography-guided CNB is a useful, yet minimally invasive diagnostic tool to identify the histological subtype of lung adenocarcinoma. It not only helps in planning the surgical and adjuvant management of the patients, but also guides the patient-prognosis.

## Introduction

Lung cancer is a leading cause of mortality worldwide, regardless of any gender predilection. The most common type of lung cancer is adenocarcinoma (ADC), with subtypes lepidic, acinar, papillary, micropapillary and solid ADC [1-3]. Seventy percent of the lesions diagnosed at the time of presentation are irresectable and at an advanced stage [1,3]. Although computerized tomography (CT), utilizing a low dose and a high resolution, aids in the easy and early diagnosis of pulmonary lesions, but neoplastic lung lesions require further work-up to reach a definitive diagnosis. Techniques including bronchoscopy, transthoracic needle aspiration and surgical biopsy are most commonly utilized to not only identify but also to differentiate between malignant and benign lesions [1,2]. Histopathological assessment of these neoplastic lesions obtained commonly via CT-guided core needle biopsy (CNB) or fine needle aspiration biopsy (FNAB), not only ensures their comprehensive classification but also assists in performing their detailed genetic analysis [1,2]. CT-guided CNB is generally preferred over FNAB because of a high volume of tissue procured via the former, thus having a diagnostic yield of 91% [2,4]. The elaborate and unique genetic makeup of each pulmonary neoplasm along with their types and subtypes obtained via the aforementioned techniques, has brought about specific targeted molecular therapies that are helpful in curing these pathologies [4]. For instance, pemetrexed is more effective in treating ADC than squamous cell carcinoma [1]. Similarly, the micropapillary and solid subtypes of ADC having a high recurrence rate, diagnosed properly via CT-guided CNB and completely resected via a lobotomy results in complete remission of the disease [2,5]. Thus, a good prognosis of each tumor type and subtype is also dependent upon a prompt and an efficient image-guided biopsy technique.

The rationale of our study is not only to understand the significance of CT-guided CNB in diagnosing pulmonary neoplasia but also to ensure the correct acquisition of a biopsy of inaccessible and irresectable lesions. The correct categorization of each pulmonary neoplasm into types and subtypes obtained via this technique also falls under the umbrella of our study. To the best of our elaborate literature search and study using Medline and Google Scholar, this is the first study on the topic from Pakistan.

## Materials and methods

This retrospective, observational study was conducted at the Department of Pathology, Aznostics - the Diagnostic Center, Lahore, Pakistan in July 2023, after ethical consideration, reference# 09/TDC/ERB/2023, dated July 05, 2023. We analyzed the records of 73 patients who underwent computerized tomography-guided core biopsy (CTCB) for pulmonary masses from January 01, 2019 to June 30, 2023, irrespective of their age and gender. The inclusion was consecutive and sampling was non-probability, convenience sampling.

Information for data set of patient’s demographics, location, and laterality of the lesion, histopathological type and histopathological subtypes were collected and analyzed via Google Form (Google Inc., Mountainview, CA) and Statistical Package for Social Sciences (IBM SPSS Statistics for Windows, Version 24, released 2016; IBM Corp., Armonk, New York, United States) respectively, which was later sent to a statistician for descriptive analysis. Frequency and percentage were calculated for categorical data and mean with standard deviation was computed for continuous data.

## Results

The mean age of 73 patients was 64.88 ± 11.39 years, with predominant male population (Table 1).

**Table 1:** Gender distribution of included patients, where N = 73.

Sr. No	Gender	Number of Cases, n	Percentage Occurrence, n/N
1.	Male	45	61.64%
2.	Female	28	38.36%

The superior lobe of the lungs is the most common site of malignancy in our study, with right-sided predominance (Tables 2-3).

**Table 2:** Sites of pulmonary malignancy, where N = 73.

Sr. No	Site of Malignancy	Number of Cases, n	Percentage Occurrence, n/N
1.	Upper lobe	42	57.53%
2.	Lower lobe	17	23.29%
3.	Middle lobe	1	1.37%
4.	Apex	1	1.37%
5.	Not specified	12	16.44%

**Table 3:** Laterality of pulmonary malignancy, where N = 73.

Sr. No	Side of Malignancy	Number of Cases, n	Percentage Occurrence, n/N
1.	Right	43	58.90%
2.	Left	30	41.10%

Seventy-three cases of ADC had the following subtypes (Table 4). There were cases where more than one pattern was identified. CT-guided core biopsy could identify histopathologic subtypes of malignancy in 55 cases, revealing a diagnostic yield of 75.34%.

**Table 4:** Subtypes of adenocarcinoma lung noted on computerized tomography-guided core biopsy, where N = 91.

Sr. No	Subtypes of Adenocarcinoma	Number of Cases, n	Percentage Occurrence, n/N
1.	Acinar	47	51.65%
2.	Solid	16	17.58%
3.	Lepidic	13	14.29%
4.	Micropapillary	8	8.79%
5.	Not specified	7	7.69%

## Discussion

Histological subtypes of lung ADC play an important role in planning surgery and as a prognostic predictor. Common means of biopsy for lung lesions suspicious of malignancy include FNAB, computer tomography-guided CNB and ultrasound guided core biopsy. Our study aimed to identify subtypes of lung ADC through CT-guided CNB.

An insight into the existing English scientific literature shows the higher accuracy of CT-guided CNB over other diagnostic modalities. A study conducted by Yao et al. showed CNB performed after FNAB was helpful in identifying the lesions, hence enhancing diagnostic yields for CNB. However, among these two procedures the best procedure depends on the local availability of resources and expertise in diagnostic techniques [6]. Similarly, a study in 2013 by Ferretti et al. showed that CT-guided transthoracic needle biopsy (TTNB) provides an accurate histological sub-typing of lung adenocarcinomas and gives good quantity of deoxyribonucleic acid (DNA) for genetic analyses [7]. In accordance with the results from the study analysis performed in University of Pittsburgh Medical Center by Schneider and his colleagues, CNBs are better than FNABs to get adequate tissue sample for molecular testing of lung adenocarcinomas [8]. Yet another study conducted at University of Sarajevo, Bosnia and Herzegovina by Beslic and his colleagues showed FNAB samples were adequate for definitive diagnosis in 79.60% and inadequate in 20.40% of patients, while CNB samples were found adequate in 96.85% and inadequate in 31.50% of patients [9].

Our study comprised 61.64% male and 38.36% female population indicating more males than females, similar to the study conducted by Zhang et al., which revealed that 71.25% of their patients were males [1]. These results are also in line with those of a-study conducted in the United States having a greater number of males than females [5]. The mean age of presentation in our study was 64.88 ± 11.39 years, similar to the findings of Zhang et al. who reported a mean age of 61.53 ± 8.94 years in males and 61.53 ± 8.94 years in females [1].

A study conducted by Chen et al. found 57.6% patients diagnosed with ADC by combined CNB and fine needle aspiration cytology (FNAC) [4]. Among subtypes of adenocarcinoma, acinar being 47.96% was the most common histological subtype. Similarly, a study by Zhang et al. conducted in China found 25 patients surgically diagnosed with acinar ADC out of which 24 were confirmed by biopsy [1]. The results of our study coincide with the study conducted by Kim et al. according to whom the most common histological subtype of lung malignancies were acinar (n=203) and lepidic (n=147) [5].

In 2010 Yoshizawa along with his team retrospectively reviewed 514 patients who had stage 1 lung ADC and underwent lobectomy with mediastinal lymph node dissection. They classified these patients according to the newly proposed classification by International Association for the Study of the Lung Cancer, American Thoracic Society and European Respiratory Society (IASLC/ATS/ERS) and showed that the histological subtyping of lung ADC along with tumor invasive size provides prognostic value for the identification of patients requiring adjunctive therapy [10]. Similarly, a study was conducted in New York, United States between 2008 and 2015 where they compared the outcome of Stereotactic Body Radiotherapy (SBRT) in different histological subtypes of lung ADC. Core biopsies were performed to label the cancerous nodules according to the 2015 WHO classification as lepidic, papillary, acinar, micropapillary, and solid sub types. The study showed that lung ADC nodules with micro-papillary and solid patterns show worse outcomes and more chances of local and metastatic progression, proving that histological sub typing helps in knowing the prognosis of lung ADCs and treatment choices in the early stages [11].

The most common site of lung malignancy was the upper lobe (57.53%) while the least site was the apex and the middle lobe. A study conducted in Austria by Klikovits et al. found that upper and middle lobe involvement of tumors was 66% while lower lobe being 33% affected [12].

According to our study, right-sided involvement of lung tumors was 58.91% and left sided involvement was 41.10%. Some studies showed that right sided lung tumors occurrence is more than that on left side. Our findings are in support of the above-mentioned findings of an Austrian study [12,13].

The diagnostic yield of CT-guided core biopsies in our study is 75.34%. A Chinese study conducted in 2022 had diagnostic yield of 96.30% in Rose group and 86.10% in non-Rose group [2]. Zhang and his co-workers in their study conducted in 2022 reported diagnostic yield of 90.65% in group A and 94.24% in group B of people with lung tumors [1]. A study conducted by Tsai and his team retrospectively reviewed and compared the results for histological subtyping of lung ADC with preoperative biopsy and after surgical resection in 318 sub-solid nodules. The study showed that preoperative biopsy was 64% concordant in determining the histological sub type, especially with tumor size less than 2 cm, predicting high-grade ADC and predicting the micropapillary and solid sub types. Hence, preoperative CT-guided CNB provides supplementary aid in classifying according to the histological subtype of lung ADC [14]. Heerink et al. have studied the complication rates of CT-guided core and fine needle biopsy in their meta-analysis and considered the rates of minor complications acceptable. Minor complications, like pneumothorax not requiring intervention, transient hemoptysis and ground glass opacity indicative of pulmonary hemorrhage were common in CT-guided biopsies [15].

Limitations

There are several limitations in our studies. First, the sample is small but our study will help in future prospects. Second, the post-biopsy complications are not discussed. Third, there is no mention of inter-observer and substantial bias. However, our study describes the importance of preoperative biopsy, diagnosis of histological subtype for prognosis prediction and treatment planning.

Clinical recommendations

Based on the collective evidence from recent research, clinicians should opt for CT-guided CNB, encompassing both FNAB and core biopsy, as the preferred method for obtaining tissue samples in patients suspected of having lung malignancies. It is imperative for clinicians to accurately identify the histologic subtypes of adenocarcinoma, as this information carries significant weight in treatment decisions and prognostic assessments. In all cases, a multidisciplinary approach involving radiologists, pathologists, and oncologists is paramount for ensuring optimal patient management and treatment outcomes.

## Conclusions

Surgical treatment and prognosis of lung malignancies are dependent on diagnosis of histological subtype of malignancy. Our study highlights the central role played by CT-guided CNB in timely diagnosis of histological subvariants and helps surgeons in customized and individualized pre-operative planning and treatment strategy for their patients. Histological subtyping for lung ADC using CT-guided CNB is reliable and safe practice.
